# Reaching everyone: school nurses’ experiences of including refugee and migrant students in the extended school-based HPV vaccination programme in Sweden

**DOI:** 10.1177/17449871251329001

**Published:** 2025-06-26

**Authors:** Ylva Odenbring, Lisa Lindén

**Affiliations:** Professor of Education, Department of Education and Special Education, University of Gothenburg, Gothenburg, Sweden; Associate Professor, Division of Science, Technology and Society (STS), Chalmers University of Technology, Gothenburg, Sweden

**Keywords:** HPV, inequality, migrant, public health, refugee, school nurses, vaccination

## Abstract

**Background:**

In Sweden, providing a free-of-charge national child vaccination programme is part of national public health work to promote health and prevent illness. Yet Sweden is no exception when it comes to systematic societal inequality. Research worldwide has shown that childhood vaccination coverage is lower among refugee and migrant children than among non-migrant children.

**Aim:**

The aim of this study is to explore how school nurses working in one of Sweden’s largest regions reflect on their strategies and experiences of including children with refugee or migrant backgrounds in the school-based extended HPV vaccination programme.

**Methods:**

The study draws from semi-structured individual interviews with 21 school nurses. Analysis drew on Braun et al’s (2011) four contextual dimensions: 1) the situated context; 2) the professional context; 3) material contexts; 4) external contexts. Thematic analysis was undertaken (Braun and Clarke, 2006; Clarke and Braun, 2013).

**Results:**

Three themes were identified: 1) social and economic deprivation; 2) ways of communicating; 3) gratitude. According to the school nurses, mapping the families’ social situation and building trusting relationships are essential. Providing written information about the vaccination in diverse languages and/or involving an interpreter are also important strategies to reach refugee and migrant parents. Despite the families’ often marginalised position, the children and their parents favour the HPV vaccination, which could be interpreted as vaccine confidence.

**Conclusions:**

Meeting the needs of children and families with refugee or migrant backgrounds requires that school nursing practice take a holistic perspective. The study contributes new insights regarding these issues.

## Introduction

One central element of public health in Sweden is that everyone should have the same opportunities for achieving good and equal health (Public Health Agency of Sweden, 2024f). Providing a national child vaccination programme is one of the central elements of Swedish preventative and public health work for children (Public Health Agency of Sweden, 2024f). These vaccinations are voluntary and offered free of charge (Public Health Agency of Sweden, 2024b). The present paper focuses on the extended school-based human papilloma virus (HPV) vaccination programme that is offered to all Swedish students in the fifth school year of primary school (students aged 11 years). The programme’s aim is to prevent a range of HPV-related cancers. In August 2020, this vaccination programme was extended to also include boys as well as girls (Public Health Agency of Sweden, 2024e).

Sweden is no exception when it comes to inequality and systematic differences between societal groups. Systematic differences in equal health include such factors as gender, sexuality, gender identity, refugee or migrant background, disabilities, and age (Grandahl et al., 2019; Grandahl and Nevéus, 2021; Odenbring and Lindén, 2023; Public Health Agency of Sweden, 2024a). For instance, research has shown that being a migrant, despite the fact that this is a heterogenous group, entails increased risk due to, for example, poorer access to healthcare and lack of knowledge about how to access contraceptives that promote young people’s sexual and reproductive health (Public Health Agency of Sweden, 2020). There is limited research in the area of sexual and reproductive health focusing on young people with a refugee or migrant background (Metusela et al., 2017; Public Health Agency of Sweden, 2020). The present paper hopes to contribute new knowledge about such issues by investigating the extended school-based HPV vaccination in Sweden in relation to young people from a refugee or migrant background.

The aim of the paper is to explore how school nurses working in one of Sweden’s largest regions reflect on their strategies and experiences of including children with a refugee or migrant background in the school-based extended HPV vaccination programme. The following research questions have guided the investigation:

(1) What issues do the school nurses address regarding inequalities?
(2) What strategies do the school nurses use in their professional work to reach and inform families with diverse language backgrounds?
(3) How do the school nurses perceive the extended vaccination programme is received by refugee and migrant students and their parents?

The present study draws on the United Nations High Commissioner for Refugee’s (UNHCR) definition of refugees and migrants. Refugees include people who are forced to flee armed conflict or persecution and need sanctuary in another country. Migrants are people who migrate mainly to improve their lives by, for instance, finding work, securing better educational opportunities or achieving family reunification (UNHCR, 2024).

## Literature review: Childhood vaccinations among refugee and migrant children

Studies worldwide have shown that childhood vaccination coverage is lower among refugee and migrant children than among non-migrant children (Abdi et al., 2019; Anuforo et al., 2022; Aragones et al. 2016; Charania et al., 2018; Khodadadi, 2020; Koa et al., 2019; Nasiri et al., 2023; Netfa, 2020). Therefore children’s foreign-born status is a risk factor for being under-immunised. Researchers have identified similar explanations for why the vaccine rate is lower among refugee and migrant children. Lack of knowledge about vaccines and vaccinations, mistrust in vaccinations, mistrust in authorities, language barriers, and low parental participation have been identified as some of the main barriers (Abdi et al., 2019; Anuforo et al., 2022; Khodadadi, 2020; Nasiri et al., 2023; Wilson et al., 2021).

Research focusing on HPV vaccinations and vaccination coverage has shown similar patterns. Research in the United States has shown that negative vaccine perceptions, lack of HPV vaccine knowledge, and lack of provider information are barriers among Latino migrant parents (Aragones et al., 2016). Aragones et al. (2016) argued that Latino parents would benefit from increased provider information and tailored vaccine information. Similarly, Koa et al.’s (2019) interview study with East African mothers in the United States, shows that mothers had limited knowledge about HPV vaccines. However, they had abundant information or misinformation about general vaccine side effects. The authors argued that providers need to find ways to connect to mothers. To gain trust and increase vaccine uptake, the authors suggested that pointing out the shared goal of protecting their child’s health and drawing on various trusted sources in the community are important strategies for influencing parents’ vaccine decision-making.

A recent Canadian study shows that newly arrived migrants, despite their lack of knowledge about HPV, had a strong desire to receive the HPV vaccine (Wilson et al., 2021). When the vaccination was recommended by a physician, this had a particularly positive impact on the migrants’ decision. Wilson et al. (2021) underlined the importance of ensuring that newly arrived migrants receive language-appropriate information about the vaccination.

Finally, in a systematic review by Graci et al. (2024) on barriers to and facilitators of accessing HPV vaccination in migrant and refugee populations, findings such as lack of knowledge and information are summarised using the notion of health literacy. The authors highlight the crucial role of accessible and comprehensible health education in promoting vaccine programmes; HPV vaccine adherence was negatively impacted by both low health literacy and lack of interest and motivation due to other health problems, by comparison after receiving adequate information on HPV parents’ willingness to vaccinate their children increased.

Although not focused specifically on HPV vaccination, one contemporary interview study with refugee mothers in New Zealand indicates that mothers had positive perceptions of the national vaccination programme (Charania, 2023). Trust was a key factor in their decision. This trust could be referred to as vaccine confidence, that is, mothers’ trust in vaccines, the health system and health providers’ recommendations. Charania (2023) concluded that health providers have a central role to play in gaining mothers’ trust. Moreover, qualified interpreters and appropriate information are important in overcoming language barriers and promoting parents’ sense of inclusion.

Given our focus on issues surrounding social inclusion and school nurses’ HPV vaccination work to include students with a refugee or migrant background in the extended school-based HPV vaccination programme, the present study aims to contribute new insights into the discussion on social inclusion, HPV vaccination and public health in a Swedeish setting.

## Methodology

The study is part of a national research project funded by a grant from the The Swedish Research Council for Health, Working Life and Welfare. The aim of the project is to investigate implementation of the extended HPV vaccination programme in Swedish primary schools. The study draws on interviews with: policy actors at the national level, policy actors in one of Sweden’s largest regions, school nurses within the same region, students in the fifth year of primary school in the same region and parents/guardians. The policy actors include physicians, school nurses, investigators, a communicator, and a health economist. Observations of the vaccination process at two different primary schools within the same region have also been conducted. Approximately 20% of the Swedish population lives in the investigated region (Statistics Sweden, 2024). The present paper draws on individual interviews with school nurses.

An ethical review application was submitted to the Swedish Ethical Review Authority 2020-06051 before the study could be conducted. The board approved the application in December 2020. After approval, potential participants were contacted and informed about the study.

A total of 21 school nurses gave their written consent to participate in the study. Before giving their consent, all school nurses were informed about the study aim and ethical guidelines. Following national ethical guidelines on confidentiality, all participating school nurses have been anonymised and are referred to as ‘school nurse’ in the study. The names of municipalities and school districts are also pseudonymised (Vetenskapsrådet, 2017).

The authors of the present paper have shared responsibility for conducting the interviews with the school nurses. AUTHOR 1 conducted 11 interviews and AUTHOR 2 conducted 10 interviews. All interviews with the school nurses were conducted in 2021 during the COVID-19 pandemic. Due to national restrictions, all interviews were conducted online via video calls. One positive outcome of this was that it enabled us to extend the study and include more school nurses than we had initially intended. Another positive outcome was that we managed to cover a much larger geographic area in the region. Demographically, the present study covers school nurses working in primary schools in a greater metropolitan area, mid-size and small towns, as well as rural areas. Online interviews also involve challenges regarding the social distance they create. In some cases, physical interviews may have been easier and more comfortable for the school nurses.

The interviews were semi-structured in character, which means that we prepared interview questions within a thematic framework, but also that we asked follow-up questions based on what emerged during the interview (Alvesson, 2011). The interview guide covered: the schools’ demographics; implementation of the extended vaccination programme; the school nurses’ practical work with the vaccinations; strategies for reaching out to and informing the students and their guardians about the new vaccination policy; and informing about HPV, sex, sexuality, and cancer. All interviews were audio recorded and fully transcribed. Given that the study includes school nurses working in Swedish public schools, all interviews were conducted in Swedish. The extracts selected for the present paper were translated into English during the process of writing the paper. All translations have been carried out by the authors of the present paper. In the narratives, the school nurses refer to and use the term *refugees* [flyktningar in Swedish] and the term *immigrants* [invandrare in Swedish] when referring to the students and their families. In some cases, they may have used the terms interchangeably. Given the importance of authenticity, we have directly transcribed and translated what the school nurses expressed. As addressed earlier in the paper, we use the terms refugees and migrants when analysing and discussing the results. The same applies to the term *segregation*: It was both a theme raised by the school nurses and a term they used to talk about specific areas within their municipality, town and/or city.

Due to its specific focus, the present paper draws on interviews with school nurses with professional experiences of meeting students and families with a refugee or migrant background. To analyse and position the school nurses’ narratives in relation to their reflections on social inclusion, inequalities and segregation, the study has been inspired by Braun et al.’s (2011) contextual dimensions. Braun et al. (2011) stated about four broad contextual dimensions: (1) situated contexts refer to those aspects of context that are linked to the school’s demographics, such as geographical location and intake; (2) professional context: the professional culture refers to professionals’ values, commitments and experiences as well as ‘policy management’ in schools; (3) material contexts refer to staffing, budget, buildings and infrastructure and (4) external contexts refer to legal requirements and responsibilities. Although these contextual dimensions are categorised and grouped, they often overlap and interconnect (Braun et al., 2011).

Although Braun et al. (2011) applied these contextual dimensions to the context of teachers in schools, we have found that they are also suitable when analysing school nurses’ work in school. In the present study, we have interpreted and applied the contextual dimensions in the following way: Situated context refers to schools dealing with segregation and social exclusion issues. Professional culture refers to school nurses’ values and commitments as well as their experiences with including all students in the vaccination programme. The material context refers to the given infrastructure of school nurses’ work, that is, the strategies and methods school nurses use to reach out to all students and families. The external context refers to national policies, that is, the national decision to include all children in the school-based HPV vaccination programme, and this context will be discussed in the concluding section, whereas the other three contexts will be more present in the analysis of the empirical data.

## Results

The data were analysed thematically (Braun and Clarke, 2006; Clarke and Braun, 2013). Thematic analysis has provided a useful method of analysing recurrent patterns in the data and of defining and naming themes. In the present study, the thematic analysis included five main steps; the authors jointly: (1) read all the transcripts multiple times to become familiar with the data, (2) coded and anonymised the data, (3) discussed and analysed recurrent themes, that is, searching for themes, (4) analysed and organised the data into main themes and, finally, (5) theorised the data. Three main broad themes were discerned in the analysis, and the results will be presented under three main headings and in the following order: (1) social and economic deprivation, (2) ways of communicating and (3) gratitude.

### Social and economic deprivation

In the study, inequalities are described by school nurses working in urban as well as rural areas. Inequalities will be addressed by regarding different social and economic factors and framed from the situated context of the school nurses’ workplace (cf. Braun et al., 2011). One of the school nurses working in a rural area discussed the increased segregation in the village. Due to the war in Syria, a large number of Syrian refugees fled to Sweden in 2015, many of whom have settled in the village. According to the school nurse, this resulted in increased segregation in the village.

School nurse, school district T: ‘*Segregation has increased in Timber town, it has really increased. There were quite a lot of immigrant families living here before and we received many refugees during the refugee crisis [referring to the war in Syria], so it’s become quite demanding. On the other hand, we’re used to working with these issues. /…/. Now there are more families that are struggling, and I suffer with the children*.’

The school nurse from school in district T continued: ‘*We have to fight many problems, it’s socioeconomically hard here, but I like it [the challenging job] because then you know they really need you*’. The picture that emerges in the narratives is of a rural community that has become more ethnically diverse, and many of the refugee families are struggling financially and socially. According to the school nurse, she has an important role to play in supporting vulnerable families.

The topic of language barriers also emerged from the school nurses’ narratives. In one of the investigated urban schools, one school nurse reflected on this issue.

School nurse, school district Y: ‘*Many different languages are spoken among the students at this school, and there are many parents, I think, who just tick yes or no. Well, they either trust and follow the system or they don’t, I guess. /…/ Many parents don’t speak Swedish and well…*’

In the extract above, the situated context is contextualised in relation to the students’ home environment. According to the school nurse, many parents do not speak Swedish, which creates difficulties for them in their everyday lives. In her experience, these parents simply tick ‘yes’ or ‘no’ on the consent form for the HPV vaccination, without further communication with her. Along the same lines, the school nurse working in school district T discussed how the situated context has impacted collaboration between the school and the parents.

School nurse, school district T: ‘*The thing I find challenging is the collaboration with the parents. The children are no problem, we work hard with the children. The difficult part is the collaboration with parents who are excluded from society, i.e., who are experiencing long-time unemployment and have not learned the language [Swedish]. It was not that long ago Swedish for immigrants was made mandatory, so I still meet parents who’ve never attended the Swedish for immigrant courses, and unfortunately they’re stuck in long-term unemployment. The families live in overcrowded housing. As a school nurse working for a long time at the same school, you get to know the families quite well. I’ve met older siblings, which means I’ve met the parents many times. It’s sometimes quite challenging, but I’ve become more open with the parents. When you’ve created a relationship with the parents, you can encourage them to learn the language and tell them how important that is. It’s not a problem for me to tell them this when I know them. My work is built on continuity and trust. There are very few parents who get angry, to be honest. If you say things in the right way, there’s normally no problem. They understand that I care about their children*.’

Here, the situated context is framed by different exclusion processes. Long-term unemployment, overcrowded housing, and lacking language skills are mentioned. The professional context is framed in relation to the importance of trust and continuity. According to the school nurse quoted above, encouraging parents to learn Swedish is central, and this also applies to the vaccination decision (knowing that they understand what they are agreeing to). Still, one critical question one might ask is whether the parents are truly unaware of the importance of language skills. Previous studies have suggested that refugee and migrant parents are aware of the importance of learning Swedish. One study raised this issue from a different perspective, showing that refugee and migrant parents stress the importance of their children learning Swedish in preschool (Sandell and Nyrén, 2013). From the parents’ perspective, learning Swedish is important for their children’s future education, ability to manage schoolwork, and academic outcomes. In the next section, we elaborate on how the school nurses, in their professional work, try to find ways to reach parents with diverse language backgrounds.

### Ways of communicating

When the school nurses talked about ways of reaching refugee and migrant parents with information about HPV vaccination, their narratives were framed by both the professional and material context (cf. Braun et al., 2011).

YO: ‘*Do you have information about the vaccination programme in several languages?*’

School nurse, school district T: ‘*Oh yes, in every language that is needed. Somali, Arabic, and other languages. I usually also send the information in Swedish, both in Swedish and the language spoken in the home environment. Maybe the parents speak Arabic, but the children normally speak Swedish very well, they learn Swedish very quickly.*’

YO: ‘*Right. How do you find out about what first language is spoken in the children’s home environment?*’

School nurse, school district T: ‘*I receive this information when the children are six years old and begin preschool class. I meet the families at this point.*’

In this case, the professional context is framed in relation to historical knowledge about the families and their first language. The material context includes written information about the vaccination in diverse languages. Similar routines regarding written information in diverse languages were described by several other school nurses.

School nurse, school district C: ‘*We have quite a lot of immigrant families, so I make copies. I’ve made copies in Arabic, Somali, and some other languages. The Public Health Agency of Sweden has this information, and the students tell us what language they want the information in. The students speak better Swedish [compared to their parents], and the parents’ Swedish is normally deficient.*’

The topic of written information in the families’ respective first languages is recurrent in the school nurses’ narratives. The written information is accessed and received via the Public Health Agency of Sweden. As part of promoting public health, the agency offers school nurses procedural support and written information about HPV in various languages. What varies, however, is the professional context, that is, how school nurses find ways to map the different languages. One of the school nurses talked about making an inventory of the languages spoken in the home environment so she can send vaccination information that is accessible for everyone.

School nurse, school district O: ‘*I’ve made an inventory of all languages spoken among the students. I asked the class teacher to inform me which students speak another language, and I sent the information both in Swedish and in the mother tongue. /…/ It was information in Somali and all Indian languages. I made a kit and sent it by mail. I sent it to all students in my school district, both the town school and rural school, and I’ve received a quick response.*’

This school nurse’s narrative is framed in relation to a professional context that involves collaboration with schoolteachers in the fifth school year and asking them for help in mapping the diverse languages spoken by the students and their families. The material context includes information about the vaccination and a letter of consent sent by mail to the parents. However, sometimes written information is not the appropriate way to reach the parents, as mentioned by a school nurse who works in school district M.

‘*If you search online there is a lot of HPV information in diverse languages. One of my students has a Somali background and I sent the information to her muom. I never received a response, so I booked a meeting with an interpreter instead and informed her orally about the vaccination. During this meeting, she told me she’s illiterate, well she can only read a little, and then written information doesn’t work, you know what I mean?*’

‘*I always have an interpreter who explains what it is; if it’s important do they understand what they are approving or not.*’

In the quote above, the professional and material context are framed in relation to an awareness that not all parents are able to understand written information due to lack of literacy or illiteracy. Notably, the attention is on lack of language literacy, and not on lack of health literacy, such as not knowing about HPV and HPV-related cancers. In those cases, the school nurse involves an interpreter who helps to translate the information orally. There are also other challenges in reaching parents with a refugee or migrant background, as explained by the school nurse in school district K.

‘*If you meet a child with parents from a different country who hardly speak any Swedish, or maybe it’s not that important in their culture to return consent [forms] in time and remember the deadline. In some cases, I know this will happen beforehand and then I remind them.*’

The above narrative is framed in relation to the families’ cultural background, that is, culture is given as the explanation for parents’ non-adherence to deadlines and return consent forms in time. The school nurse did not seem to be considering the possibility that the families’ social situation, such as economic hardship, may have a negative impact on parents’ ability to remember things and keep things in order. By locating the issue to ‘cultural background’, social factors, such as those related to systematic differences between groups in society, are not taken into consideration.

### Gratitude

Our data indicate that most parents, irrespective of their children’s gender, have their children vaccinated against HPV, and the school nurses reported that inclusion of boys in the extended vaccination programme has been unproblematic (Odenbring and Lindén, 2023). Moreover, the school nurses did not refer to the children’s refugee or migrant background as an obstacle, on the contrary. During the COVID-19 pandemic, when the present study was conducted, Swedish primary school children attended school as usual (Public Health Agency of Sweden, 2024c). The authorities based this decision on what was considered best for children’s short-term as well as long-term health and general well-being. As a result, the school-based HPV vaccinations were carried out as planned.

When meeting refugee and migrant parents and their children, the school nurses brought up what could be defined as optimistic experiences related to the students’ and their family’s situated context. These narratives are framed as expressions of gratitude.

School nurse, school district M: ‘*Families from other countries, well I don’t have that much experience, but they are very grateful for the vaccinations. They approve most things; you know what I mean? There is normally no problem, that’s my experience. They normally say yes, but you must explain what it is of course, that’s very important.*’

The school nurse quoted above reported that the parents had positive attitudes towards HPV vaccination. They were described as being grateful that their children had been given the opportunity to get vaccinated. Gratitude is recurrent in the school nurses’ narratives. Expressions of gratitude are also framed in relation to the families’ previous lack of opportunities to receive vaccinations in their home countries.

School nurse, school district C: ‘*We have many immigrant children at this school, and they’re normally happier about the vaccinations. Grateful. They have experienced at lot in their home countries and have not been able to get vaccinations for various reasons, like war, and then they come to Sweden, I’ve experienced that a lot. /…/ They rarely say no, they’re simply so happy that their children are getting protection.*’

The school nurse quoted above referred to the families’ previous experiences of war and gave this as an explanation for their gratitude about having their children vaccinated. Another dimension addressed by one of the school nurses is schools with different catchment areas.

School nurse, school district B: ‘*A couple of years ago I was assisting a school nurse at a school where most of the children come from another country, and they live in an economically and socially deprived area. I think 97 percent of the children are from other countries. This was during the time when only girls received the vaccine. I was so impressed that everyone was so grateful. ‘Thank you so much for the vaccination’, they said when they left [after receiving the vaccination]. That’s not really what I’ve experienced at my current school. I don’t know how to put it, but they kind of take it for granted, but at the other school it was like ‘thank you so much for this vaccination’. I’ve experienced such a difference in their behaviour.*’

The school nurse quoted above framed, and referred to, appreciation as being polite. The narrative is also framed in relation to students’ attitudes, that is, not taking everything – including access to childhood vaccinations via the school health system – for granted. Here, the students at her current school, which are predominately white middle-class children, are referred to as taking the vaccination for granted.

## Discussion

The aim of the present paper was to explore how school nurses reflect on their strategies and experiences of including children with a refugee or migrant background in the extended school-based HPV vaccination programme. Introducing the extended vaccination programme, which includes boys, has been fairly uncomplicated (Odenbring and Lindén, 2023). Nonetheless, the present study, which took a school nursing perspective, has contributed new insights into the discussion on social inclusion, HPV vaccination, and public health.

In Sweden, the HPV vaccination programme is part of the national vaccination programme for all children. This national decision frames the external context of the school nurses’ professional work in the investigated region as well as nationally (Braun et al., 2011). The present study shows that the situated context is framed in relation to narratives about the everyday struggles and inequalities the families face. The school nurses addressed long-term unemployment, overcrowded housing as well as limited skills in the Swedish language. This shapes the professional context of the school nurses’ work (Braun et al., 2011). Here, they talked about the importance of strategies, such as identifying families’ social situation, building trusting relationships, and identifying what first language is spoken in the children’s home environment. Mapping the first language is vital to being able to reach and inform the parents in the right way. Communication with the parents is framed in relation to a material context that includes written information about the vaccination in diverse languages; in some cases, due to lack of literacy or illiteracy, an interpreter must also be involved (Braun et al., 2011). The importance of interpretation and of meeting and challenging language barriers has also been addressed in previous studies (Abdi et al., 2019; Charania, 2023; Nasiri et al., 2023). What differentiates this study from previous studies is that our study shows how professionals, in this case school nurses, pragmatically do this in their professional work. Moreover, in contrast to previous studies focusing on migrant or refugee parents’ lack of knowledge about HPV and HPV transmission (Graci et al., 2024), our study demonstrates the importance of attending to questions of inequality.

The narratives also reveal a situated context framed in relation to an optimistic outlook. Despite the families’ marginalised position and the difficulties they often face, the students and their parents were described in a positive manner. Recurrent in these narratives is the experience that refugee and migrant parents have positive attitudes towards the vaccination and are grateful that their children have been given this opportunity. One possible explanation for the parents’ positive reactions is vaccine confidence, that is, trust in the vaccine, the information they have received, the health system, and the school nurse (cf. Charania, 2023). This is supported by the high levels of parental trust previously reported in Swedish studies of childhood vaccination (Byström et al., 2020), including HPV vaccination (Runngren et al., 2022). Similarly, the description of students’ reactions could also be interpreted in the light of vaccine confidence.

## Conclusion

The present study contributes new insights into the multifaced work school nurses do when meeting the needs of children and families with a refugee or migrant background. A holistic perspective is important to understanding the social dimensions of school nursing practice.

**Key points for policy, practice and/or research**

- The study contributes new insights into HPV vaccinations of refugee and migrant children – which currently is an under-researched area.
- Building trusting relationships, and mapping and identifying families’ social situation are crucial to offering the appropriate support.
- Despite the struggles many refugee and migrant families face every day, our results indicate an existing vaccine confidence.
- Disseminating the present results to school nurses and policymakers could raise awareness of refugee and migrant childrens’ specific needs and improve the vaccination programme’s existing infrastructure.
- To further investigate these issues it would be important to conduct interviews with students and parents with a refugee and migrant background. We believe this would deepen and add new insights into these issues. Different groups or individuals may have different needs, and it is vital to give them a voice. As pointed out by the Public Health Agency of Sweden (2024d), to avoid the risk of stigmatisation and segregation of certain groups, it is important to individual perspectives in mind.

## References

[bibr1-17449871251329001] Abdi I Menzies R Seale H (2019) Barriers and facilitators of immunisation in refugees and migrants in Australia: An east-African case study. Vaccine, 37(44): 6724–6729. 31537444 10.1016/j.vaccine.2019.09.025

[bibr2-17449871251329001] Alvesson M (2011) Intervjuer. Malmö: Liber.

[bibr3-17449871251329001] Anuforo B McGee‑Avila JK Toler L , et al (2022) Disparities in HPV vaccine knowledge and adolescent HPV vaccine uptake by parental nativity among diverse multiethnic parents in New Jersey. BMC Public Health, 22:195, 1–8. 10.1186/s12889-022-12573-7 PMC8800253 35093050

[bibr4-17449871251329001] Aragones A Genoff M Gonzalez C , et al (2016) HPV vaccine and Latino immigrant parents: If they offer it, we will get it. J Immigrant Minority Health, 18:1060–1065. 10.1007/s10903-015-0225-x PMC4656145 26001843

[bibr5-17449871251329001] Braun A Ball SJ Maguire M , et al(2011) Taking context seriously: Towards explaining policy enactments in the secondary school. Discourse: Studies in the Cultural Politics of Education, 32(4): 585–595.

[bibr6-17449871251329001] Braun V Clarke V (2006) Using thematic analysis in psychology. Qualitative Research in Psychology, 3(2): 77–101.

[bibr7-17449871251329001] Byström E Lindstrand A Bergström J , et al (2020) Confidence in the National Immunization Program among parents in Sweden 2016–A cross-sectional survey. Vaccine, 38(2): 3909–3917. 32057573 10.1016/j.vaccine.2020.01.078

[bibr8-17449871251329001] Clarke V Braun V (2013) Teaching thematic analysis: Overcoming challenges and developing strategies for effective learning. The Psychologist, 26(2): 120 –123.

[bibr9-17449871251329001] Charania NA (2023) “She vaccinated my baby and that’s all. . .”: Immunisation decision-making and experiences among refugee mothers resettled in Aotearoa New Zealand. BMC Public Health, 23(1349): 1–11. 37442991 10.1186/s12889-023-16266-7 PMC10347757

[bibr10-17449871251329001] Charania NA Paynter J Lee AC , et al (2018) Exploring immunisation inequities among migrant and refugee children in New Zealand. Human Vaccines & Immunotherapeutics, 14(12): 3026–3033. 30024825 10.1080/21645515.2018.1496769 PMC6343632

[bibr11-17449871251329001] Graci D Piazza N Ardagna S , et al (2024) Barriers to and facilitators for accessing HPV vaccination in migrant and refugee populations: A systematic review. Vaccines, 12(3): 256. 38543890 10.3390/vaccines12030256 PMC10975665

[bibr12-17449871251329001] Grandahl M Nevéus T Dalianis T , et al(2019) ‘I Also Want to Be Vaccinated!’– Adolescent Boys’ awareness and thoughts, perceived benefits, information sources, and intention to be vaccinated against human papillomavirus (HPV) Human Vaccines & Immunotherapeutics, 15(7-8): 1794–1802. 30481108 10.1080/21645515.2018.1551670 PMC6746528

[bibr13-17449871251329001] Grandahl M Nevéus T (2021) Barriers towards HPV vaccinations for boys and young men: A narrative review. Viruses, 13(8): 1644. 34452508 10.3390/v13081644 PMC8402923

[bibr14-17449871251329001] Khodadadi AB Redden DT Scarinci IC (2020) HPV vaccination hesitancy among Latina immigrant mothers despite physician recommendation. Ethnicity & Disease, 30(4): 661–670. 32989366 10.18865/ed.30.4.661 PMC7518535

[bibr15-17449871251329001] Koa LK Taylora VM Mohamedc FB , et al (2019) “We brought our culture here with us”: A qualitative study of perceptions of HPV vaccine and vaccine uptake among East African immigrant mothers. Papilloma Research, 21–25. 10.1016/j.pvr.2018.12.003 PMC6319298 30594650

[bibr16-17449871251329001] Metusela C Ussher J Perz J , et al (2017) ‘In My Culture, We Don’t Know Anything About That’: Sexual and reproductive health of migrant and refugee women. International Journal of Behavioral Medicine, 24(6): 836–845. 28620774 10.1007/s12529-017-9662-3

[bibr17-17449871251329001] Nasiri A Farshidi H Rezaei F , et al (2023) Perceived barriers of migrants and refugees to vaccinate their children against Measles and polio: A study in Iran. International Journal for Equity in Health, 22(1): 253. 38057773 10.1186/s12939-023-02075-2 PMC10701918

[bibr18-17449871251329001] Netfa F Tashani M Booy R , et al (2020) Knowledge, attitudes and perceptions of immigrant parents towards human papillomavirus (HPV)vaccination: A Systematic Review. Tropical Medicine and Infectious Disease, 5(58): 1–18. 10.3390/tropicalmed5020058 PMC7344469 32283644

[bibr19-17449871251329001] Odenbring Y Lindén L (2023) Gender, sex and equal health: school nurses’ strategies and experiences of including boys in the HPV vaccination programme in Swedish primary schools. Sex Education: Sexuality, Society and Learning, 23(5): 617–630.

[bibr20-17449871251329001] Public Health Agency of Sweden (2020) Migration, sexuell hälsa och hiv- och STI-prevention. En kartläggning av unga migranters sexuella och reproduktiva hälsa och rättigheter i Sverige. Report, Public Health Agency of Sweden, March.

[bibr21-17449871251329001] Public Health Agency of Sweden (2024a) Analyser av ojämlikheten i hälsa och hälsan i vissa grupper. Available at: https://www.folkhalsomyndigheten.se/om-folkhalsa-och-folkhalsoarbete/tema-folkhalsa/hur-mar-sverige/analyser-av-jamlik-halsa/#nationella (accessed 22 February 2024).

[bibr22-17449871251329001] Public Health Agency of Sweden (2024b) Barnvaccinationsprogram – Allmänt program för barn. Available at: https://www.folkhalsomyndigheten.se/smittskydd-beredskap/vaccinationer/nationella-vaccinationsprogram/barnvaccinationsprogram/ (accessed 13 January 2024).

[bibr23-17449871251329001] Public Health Agency of Sweden (2024c) Folkhälsa och barns bästa vägledande i skolbeslut under pandemin. Available at: https://www.folkhalsomyndigheten.se/nyheter-och-press/nyhetsarkiv/2023/oktober/folkhalsa-och-barns-basta-vagledande-i-skolbeslut-under-pandemin/ (accessed 16 September 2024).

[bibr24-17449871251329001] Public Health Agency of Sweden (2024d) Migranter – en heterogen grupp. Available at: https://www.folkhalsomyndigheten.se/livsvillkor-levnadsvanor/halsa-i-olika-grupper/migration-och-halsa/migranter–en-heterogen-grupp/ (accessed 20 September 2024).

[bibr25-17449871251329001] Public Health Agency of Sweden (2024e) Utredningar om nationella vaccinationsprogram. Available at: https://www.folkhalsomyndigheten.se/smittskydd-beredskap/vaccinationer/nationella-vaccinationsprogram/utredningar-om-nationella-vaccinationsprogram/ (accessed 13 March 2024).

[bibr26-17449871251329001] Public Health Agency of Sweden (2024f) Vad är folkhälsa, jämlik hälsa och folkhälsoarbete? Available at: https://www.folkhalsomyndigheten.se/en-god-och-jamlik-halsa-pa-alla-nivaer/tema-folkhalsa/vad-ar-folkhalsa/folkhalsa-och-jamlik-halsa/ (accessed 27 March 2024).

[bibr27-17449871251329001] Runngren E Eriksson M Blomberg K (2022) Parents’ reasoning about HPV vaccination in Sweden. Scandinavian Journal of Caring Sciences, 36(4): 1113–1122. 34672006 10.1111/scs.13041

[bibr28-17449871251329001] Statistics Sweden (2024) Folkmängd i riket, län och kommuner 31 december 2022 och befolkningsförändringar 2022. Available at: https://www.scb.se/hitta-statistik/statistik-efter-amne/befolkning/befolkningens-sammansattning/befolkningsstatistik/pong/tabell-och-diagram/folkmangd-och-befolkningsforandringar—helarsstatistik/folkmangd-i-riket-lan-och-kommuner-31-december-2022-och-befolkningsforandringar-2022/ (accessed 12 September 2024).

[bibr29-17449871251329001] Sandell A Nyrén L (2013) Likvärdighet i förskolan? In: Harju A Tallberg Broman I (eds) Föräldrar, förskola och skola: Om mångfald, makt och möjligheter, pp. 229–244. Studentlitteratur.

[bibr30-17449871251329001] Vetenskapsrådet (2017) God forskningssed. Vetenskapsrådet.

[bibr31-17449871251329001] UNHCR (2024) ‘Refugee’ or ‘migrant’ – Which is right? Available at: https://www.unhcr.org/news/stories/unhcr-viewpoint-refugee-or-migrant-which-right (accessed 19 September 2024).

[bibr32-17449871251329001] Wilson LA Quan AML Bota AB , et al (2021) Newcomer knowledge, attitudes, and beliefs about human papillomavirus (HPV) vaccination. BMC Family Practice, 22(17): 1–12. 33421999 10.1186/s12875-020-01360-1 PMC7797127

